# Designing a resilience-based intervention program for children with cancer and their families: a study protocol

**DOI:** 10.3389/fpsyg.2024.1419192

**Published:** 2024-09-04

**Authors:** Chantale Simard, Véronique Roberge, Maxime St-Pierre, Jacques Cherblanc, Christiane Bergeron-Leclerc, Mohamed Abdelhafid Kadri, Carl Lacharité, Samuel Bérubé, Laurie Lapointe, Valérie Faucher, Sebastien S. Dufresne

**Affiliations:** ^1^Département des sciences de la santé, Université du Québec à Chicoutimi (UQAC), Saguenay, QC, Canada; ^2^Centre Intersectoriel en Santé Durable (CISD), Université du Québec à Chicoutimi (UQAC), Saguenay, QC, Canada; ^3^Réseau Québécois de Recherche en Soins Palliatifs et de Fin de Vie, Québec, QC, Canada; ^4^Département des sciences humaines, Université du Québec à Chicoutimi (UQAC), Saguenay, QC, Canada; ^5^Département de psychologie, Université du Québec à Trois-Rivières (UQTR), Trois-Rivières, QC, Canada; ^6^Département de cancérologie, soins palliatifs et de fin de vie, Centre Intégré Universitaire de Santé et de Services Sociaux du Saguenay-Lac-St-Jean (CIUSSS SLSJ), Chicoutimi, QC, Canada

**Keywords:** pediatric oncology, family resilience, remote communities, Canada, intervention mapping

## Abstract

**Background:**

Advances in pediatric oncology have significantly increased survival rates, yet have introduced challenges in managing long-term treatment side effects. This study process introduces an interdisciplinary clinical intervention program rooted in the family resilience framework, aimed at improving well-being across the cancer trajectory for children and their families, especially those in Canadian communities far from specialized oncology centers with limited access to resources.

**Methods:**

Employing an intervention mapping approach, this program collaboratively involves patients, families, professionals, and researchers. It aims to identify vulnerability factors, establish a logic model of change, and devise comprehensive strategies that include professional interventions alongside self-management tools. These strategies, tailored to address biopsychosocial and spiritual challenges, are adapted to the unique contexts of communities distant from specialized cancer treatment centers. A mixed-methods approach will evaluate program effectiveness.

**Expected results:**

Anticipated outcomes include the empowerment of families with self-management tools and professional support, designed to mitigate biopsychosocial and spiritual complications. By addressing the specific needs and limitations of these communities, the program strives to improve the overall health and well-being of both undergoing treatment and survivorship phases.

**Discussion:**

By focusing on comprehensive care that includes both professional interventions and self-management, this initiative marks a significant shift toward a holistic, family-centered approach in pediatric oncology care for remote communities. It underlines the necessity of accessible interventions that confront immediate and long-term challenges, aiming to elevate the standard of care by emphasizing resilience, professional support, and family empowerment in underserved areas.

## 1 Introduction

In recent decades, significant progress in diagnostic tools and medical treatments has revolutionized pediatric oncology, resulting in a remarkable increase in survival rates for children facing cancer (Agence de la Santé Publique du Canada, 2024). Currently, the majority of these children will transition to adulthood. However, this progress is accompanied by long-term health challenges that affect both the survivors and their families. These adverse outcomes, influenced by diverse factors such as cancer subtypes, treatment protocols, and family dynamics, can arise during intensive therapy, persist long after treatment, or surface years later (Bhakta et al., 2017; Armstrong et al., 2014; Hudson et al., 2013; Bhakta et al., 2020; Robison and Hudson, 2014).

Estimates suggest that 60–90% of pediatric cancer survivors experience chronic health conditions (National Cancer Institute, 2024), including secondary cancers, cardiac toxicity, growth disorders, endocrinopathies, obesity, and cognitive dysfunctions (Tanner et al., 2020). These issues, coupled with psychosocial, financial, and spiritual challenges, often manifest in adulthood and are frequently overlooked in post-cancer care protocols. This situation is particularly dire in remote communities like Saguenay-Lac-Saint-Jean (Quebec, Canada), where families face the additional burden of limited local healthcare resources and lengthy commutes to specialized oncology centers, amplifying their vulnerability.

The rarity of pediatric cancer results in the necessary care and treatments being primarily concentrated in tertiary care hospitals located in urban areas (Scott-Findlay and Chalmers, 2001). Approximately one in five Canadians lives in a rural area (Statistique Canada, 2013), meaning that 20% of families with a child undergoing cancer treatment must endure prolonged separations from other family members, often lasting several weeks to months. Families living far from pediatric oncology centers face unique hardships, such as disrupted family dynamics, increased financial burden, emotional stress, and limited access to specialized care (Scott-Findlay and Chalmers, 2001; Walling et al., 2019). These families often have to manage secondary family arrangements, which can lead to significant strain (McCubbin et al., 2002). The physical impact of having to stay close to the sick child in unfamiliar environments, such as temporary accommodations, can disrupt normal sleep and eating patterns and exacerbate the side effects of cancer treatments due to long travel distances (Daniel et al., 2013). Psychologically, concerns about travel conditions, access to specialized care, and the roles of caregiving increase anxiety and stress (Walling et al., 2019; Aitken and Hathaway, 1993). The relational aspect is also strained, with families often finding it difficult to maintain social contacts and marital relationships due to long separations and the pressure of medical appointments (Scott-Findlay and Chalmers, 2001). Financially, the costs associated with travel, accommodation, and lost wages add to the burden, making it imperative for interventions to consider these multifaceted challenges (Walling et al., 2019; Daniel et al., 2013; Fluchel et al., 2014).

Addressing these health challenges is a major concern for healthcare professionals. The World Health Organization (WHO) has emphasized the importance of these outcomes, advocating for interventions that bridge gaps in childhood cancer research and care (Organisation Mondiale de la Santé, 2021). This has led to the adoption of holistic strategies focused on identifying, preventing, and mitigating the long-term impacts of cancer (Paul et al., 2024). Multidisciplinary and family-centric approaches during cancer care experience are crucial, as environmental factors play a significant role in preventing developmental issues and disabilities (Corrigan and Feig, 2004; Khan et al., 2022; Wiener et al., 2015). The diagnosis of cancer significantly impacts family dynamics, necessitating resilience to adapt and cope with adversity (Rosenberg et al., 2014; Van Shoors et al., 2015; Rosenberg et al., 2018; Rosenberg et al., 2019).

In this context, resilience emerges as a critical construct; it’s the process that refers to the “capacities in family functioning to withstand and rebound from adversity. More than surviving loss and coping with disruptions, resilience involves positive adaptation: regaining the ability to thrive, with the potential for transformation and positive growth forged through the searing experience” (Walsh, 2016a, p. 904). Walsh’s theoretical framework of family resilience provides valuable insights into how families navigate and recover from crises, highlighting belief systems, organizational patterns, and communication processes as key domains of family functioning. This framework offers a therapeutic approach for professionals aiming to strengthen family resilience. Despite the proven benefits of family resilience, there is a notable lack of research on its application in developing interdisciplinary intervention programs for pediatric cancer patients. This paper outlines the protocol for creating an interdisciplinary clinical intervention program (The Program), rooted in the family resilience framework, specifically tailored to address the risk factors contributing to the vulnerability of families in the Saguenay-Lac-Saint-Jean region.

## 2 Methods

### 2.1 Working groups

The development and refinement of The Program will heavily rely on collaboration with Working Groups (WGs) (Bartholomew et al., 2016). These groups will be composed of family members, health professionals, managers, and researchers. The WGs will play a crucial role in each of the six steps of the IM approach, ensuring that the intervention is grounded in practical insights and real-world experiences (Bartholomew et al., 2016).

WGs will be established, drawing members from the three primary participant pools. Each WG is set to comprise no more than nine individuals, ensuring focused, productive dialog. This composition will include seven healthcare professionals (spanning roles such as nurses, psychologists, social workers, physical therapists, pediatricians, spiritual care providers, and managers), one adult who was diagnosed with cancer during childhood, and one family member currently or previously involved in the pediatric cancer journey. This deliberate limitation on group size adheres to the insights of Johnson et al. (1993) and Hackman and Vidmar (1970), who underscored that smaller groups counteract the tendency toward passive involvement—a common pitfall in larger settings. By keeping groups more intimate, we aim to foster an environment conducive to active participation, meaningful exchange, and a deeper, more nuanced exploration of experiences and perspectives.

The initial step of this research endeavor necessitates at least four WG sessions. Each of these crucial meetings will be facilitated by one or more of the project’s dedicated team members. At the commencement of every session, assurances will be provided regarding the strict confidentiality of the data shared and the handling of any other potential ethical considerations. Participants will be informed that all perspectives are valuable, reinforcing that there are no “right” or “wrong” contributions.

Each meeting will conclude with reminders about confidentiality and expressions of gratitude for the participants’ invaluable contributions. Following the acquisition of written consent from the participants, all WG sessions will be audio-recorded. This ensures that detailed information shared during discussions is accessible for precise reference and analysis by the research team.

To comprehensively characterize the participant sample, socio-demographic data will be compiled through self-administered forms at the beginning of the inaugural WG session.

### 2.2 Intervention mapping approach

The intervention development process is structured according to the Intervention Mapping (IM) approach, which is divided into six key steps. Each step is meticulously designed to ensure a comprehensive and systematic progression from problem identification to program evaluation. The IM approach is inherently collaborative, involving patients, families, professionals, and researchers. It encourages the use of theoretical frameworks to address the multiple dimensions linked to the complex nature of health experiences. The process incorporates data from research, theory, and the experiences and opinions of those directly affected, ensuring a holistic and inclusive approach.

Table 1 provides an overview of each step, including the description, key activities, and expected outcomes. The first column, Step, enumerates the sequential steps in the IM process, from the identification of the problem to the evaluation of the program. The second column, Description, provides a brief explanation of each step, outlining its primary focus and objectives. The third column, Key Activities, details the specific actions and methods employed during each step, highlighting the practical and theoretical approaches used to achieve the objectives. Finally, the fourth column, Outcomes, lists the expected results of each step, illustrating how each phase contributes to the overall goal of developing a robust and effective intervention program. This structured approach ensures a clear, logical progression through the stages of intervention development, providing a solid foundation for creating a tailored, evidence-based program that addresses the unique needs of children with cancer and their families in the Saguenay-Lac-Saint-Jean region.

**Table 1:** Overview of the intervention mapping process steps, descriptions, key activities, and expected outcomes.

Step	Description	Key activities	Outcomes
Step 1: Logical model of the problem	Identify family vulnerability factors	Systematic literature review on potential biopsychosocial and spiritual risk factors associated with the experience of children with cancer and their family Systematic literature review on potential environmental risk factors related to family vulnerability associated with the experience of children with cancer and their family Descriptive qualitative approach and thematic analysis of vulnerability factors associated with geographical challenges from our previous work (Simard, 2023) Input from WGs including family members, health professionals, managers, and researchers	Comprehensive list of risk factors Logical model highlighting family vulnerability factors
Step 2: Logic model of change	Define protective factors and change objectives	Utilize results from the first step Integrate data from scientific literature and expert opinions (WG members) Establish expected outcomes for family experience and environment Specify family objectives to achieve these outcomes Select determinants (necessary protective factors) Create matrices to detail tasks and skills needed to meet objectives	Detailed logic model of change Defined protective factors and family and environmental change objectives
Step 3: Program development	Design the intervention program	Based on the change model, select interventions and strategies Use theories, evidence-based data, and practical applications Engage with WGs to incorporate practical strategies and adapt to geographical context	Evidence-based program Practical strategies tailored to family and environmental objectives
Step 4: Program’s organization, pre-test and refinement	Refine the program components	Determine the structure and components of the program considering the care context of Saguenay-Lac-St-Jean WG decisions on what (interventions), who (participants), when (timing), and how (format) Pre-test with selected families Gather feedback and analyze results Refine Program’s components considering context-specific resources and strengths WG discussions to improve the program Pre-test with selected families	Refined the program based on pre-test feedback Adjustments to enhance relevance and feasibility
Step 5: Implementation plan	Develop a plan for program rollout	Identify key stakeholders Select strategies for adoption and integration WG input to identify potential facilitators and barriers	Comprehensive implementation plan Strategies to facilitate program adoption
Step 6: Evaluation plan	Assess program implantation, effectiveness and impact	Data collection includes a registry for feasibility data, clinical assessments, and self-report questionnaires at baseline (T1) and end of the program (T2), and semi-structured interviews with families and health professionals (T3) Analyze feasibility, acceptability, and fidelity through various instruments and methods Quantitative data analysis using SPSS Qualitative data analysis using NVivo	Detailed evaluation plan Data on program implantation, effectiveness and impact Identified barriers and facilitators to participation Measured changes in family outcomes

#### 2.2.1 Step 1: logical model of the problem: vulnerability in pediatric cancer during treatment and survivorship

The first step in the IM approach involves comprehensively identifying vulnerability factors that contribute to poor outcomes for children with cancer and their families. This step is foundational for tailoring the intervention effectively. Initially, a systematic literature review will identify potential biopsychosocial, spiritual, and environmental risk factors associated with the experiences of these children and their families. Databases such as PubMed, CINAHL, PEDro, and Embase will be used, with the search strategy being carried out by the researchers in the working group.

Our previous study, which employed a descriptive qualitative approach and thematic analysis, identified vulnerability factors linked to geographical challenges (Simard, 2023). This previous work provides essential insights, particularly for understanding the specific impacts of geographic isolation on families in remote areas like Saguenay-Lac-Saint-Jean. We will gather input from WGs that include family members, health professionals, managers, and researchers. These consultations are crucial for capturing diverse perspectives and addressing the multifaceted challenges these families face.

The logical modeling of the problem is informed by the PRECEDE model (Bartholomew et al., 2016), requiring an ecological analysis of the causes, including both risk behaviors and environmental conditions. This approach helps us relate scientific literature findings to those identified in our previous study on the specific impacts of geographic isolation on families dealing with pediatric cancer. Using theoretical frameworks centered on family resilience (Walsh, 2016b) and strengths (Gottlieb, 2013), we analyze the problem further. We establish that insufficient mobilization of family resources and competencies (protective factors) can render families vulnerable, compromising their well-being. Vulnerability occurs when a traumatic family experience (TFE) presents more risk factors than protective factors available to counter them (Walsh, 2016a; Delage, 2008).

The logical model details risk factors from both the child and the family’s experience and their immediate environment, including healthcare, services, and social networks. These risk factors are sub-categorized according to Walsh’s domains of family functioning: communication processes, organizational patterns, and belief systems. This comprehensive model highlights the biopsychosocial, spiritual, and environmental risk factors affecting children with cancer and their families, forming the foundation for subsequent IM steps. Integrating these elements ensures the intervention is both evidence-based and contextually relevant, enhancing its effectiveness (Bartholomew et al., 2016; Bartholomew and Mullen, 2011).

#### 2.2.2 Step 2: logic model of change: prevention and management of health complications during cancer treatment and survivorship

The second step in the IM approach will involve creating a logic model of change to strengthen the resilience of families accompanying children with cancer. This step, as described by Bartholomew et al. (2016), will determine “what the health promotion program is intended to accomplish” (p. 286). After identifying the risk factors causing family problems in the first step, this second step will map out the desired change process for the intervention program.

This phase will be conducted inductively through the second meeting with the WGs. The process will be divided into several key activities. Initially, we will define the expected outcomes concerning the family experience and environment, aiming to enhance the overall well-being of children with cancer and their families by addressing specific biopsychosocial and spiritual challenges. Following this, we will set specific family objectives to achieve these desired outcomes, focusing on improving family resilience, coping strategies, and support systems.

Next, we will identify the necessary protective factors or strengths that will enable families to achieve their objectives, such as emotional support, effective communication, and robust social networks. To operationalize these objectives, detailed matrices will be created to outline the specific actions, strategies, and competencies required to achieve the family and environmental goals (Bartholomew et al., 2016). These matrices will provide a clear roadmap for implementing the intervention, specifying the tasks and skills needed to strengthen family resilience.

The protective factors identified in our previous study on the impact of geographical isolation (Simard, 2023) will be crucial. These factors, integrated with empirical and theoretical data and insights from the WGs, will inform the development of two primary matrices (Bartholomew and Mullen, 2011). The first matrix will focus on the expected family protective factors, while the second will address the family environment, including healthcare, services, and social networks. Both matrices will aim to enhance the resilience of families accompanying children with cancer by detailing specific objectives, actions, strategies, and skills necessary for achieving the desired outcomes.

By following these sub-steps, we will create a comprehensive logic model of change, which will provide a structured and evidence-based framework for the intervention (Bartholomew et al., 2016). This model will ensure that the program is designed to effectively support families, leveraging their strengths and addressing their vulnerabilities to improve their overall well-being and resilience. This methodical approach will not only define the intervention’s objectives but also outline the practical steps needed to achieve them, ensuring that the program is both theoretically sound and practically applicable (Bartholomew et al., 2016; Bartholomew and Mullen, 2011).

#### 2.2.3 Step 3: development of an interdisciplinary intervention program for children with cancer in a real-care setting

The third step will involve developing the logic model of the intervention program (The Program) (Bartholomew et al., 2016). This phase will be conducted during the third session with the WGs. This step requires analyzing and targeting the most effective empirical data, theoretical insights, and exemplary interdisciplinary practices available. It will also involve considering interdisciplinary and family interventions already implemented or suggested by all health professionals and participating family members. Additionally, a systematic literature review will be conducted to identify the best evidence-based interventions, with the search strategy being carried out by the researchers in the working group.

The goal of this step is to identify interventions that best achieve the family and environmental objectives set in the previous step. The Program will aim to prevent child and family vulnerability and optimize their well-being and quality of life. The change supported by The Program is primarily driven by strengthening family resilience, as proposed in the Family Resilience Framework (Walsh, 2016b) and by adopting a strength-based care approach centered on the child and family (Gottlieb, 2013; Gottlieb and Gottlieb, 2017).

During this phase, a summary table will be created. For each family and environmental objective identified in the second step, the table will present more specific goals to facilitate their achievement and associate each with potential interdisciplinary interventions. Given that a single intervention can address multiple objectives simultaneously, some interdisciplinary interventions will be linked to several objectives.

To ensure the interventions are comprehensive and effective, we will incorporate insights from exemplary practices, empirical research, and theoretical frameworks (Bartholomew et al., 2016). This approach will ensure that The Program is not only theoretically sound but also practically applicable, addressing the specific needs and vulnerabilities of families accompanying children with cancer.

By following these sub-steps, we will develop a detailed and actionable intervention program designed to strengthen family resilience and improve the overall well-being and quality of life for these families. This methodical approach will ensure that the program is both evidence-based and contextually relevant, providing a robust foundation for the subsequent steps in the IM process.

#### 2.2.4 Step 4: creation of the interdisciplinary intervention program for children with cancer in real-care contexts, anchored in the family resilience framework

The fourth step will involve pre-testing and refining the structure and components of The Program (Bartholomew et al., 2016). This phase will be conducted during the final session with the WGs. The goal is to determine the what (e.g., relevant and realistic interdisciplinary interventions to achieve the set objectives), who (e.g., children and families undergoing the accompaniment experience, health professionals, managers, and researchers), when (e.g., timing, frequency, duration of each intervention), and how (e.g., format, components, steps, activities) of The Program.

During this step, we will utilize a summary table to guide the discussions. The WGs will identify and define the interventions required to meet the family and environmental objectives established in the previous steps. This will include determining specific actions, strategies, and competencies needed for each objective.

To ensure that the intervention is appropriately tailored to the varying levels of family vulnerability, we will introduce a stratification of intervention intensity. This stratification will range from self-management support to a comprehensive interdisciplinary intervention plan. The method and tool for stratification will be determined by the team, selecting from existing tools such as the Psychosocial Assessment Tool (PAT) (Pai et al., 2008; Kazak et al., 2015). This approach allows the intensity of the intervention to be adapted to the level of vulnerability and specific needs of each family.

The Program will include a variety of strategies or tools designed to achieve the family and environmental objectives identified. These strategies will be categorized according to three major areas of interdisciplinary activity. The first category will focus on the training and clinical support of health professionals regarding pediatric oncology, including initiatives such as training programs, mentorship, and ongoing education. The second category will be oriented toward care management, incorporating strategies like grouping families with similar experiences, ensuring continuity of care by dedicated staff, and providing materials similar to those used in specialized oncology centers. The third category will relate to practice, involving tools and resources such as family guides, follow-up sheets, tools for assessing psychosocial needs, and summaries of child and family records.

By refining The Program through this structured approach, we will ensure that the intervention is comprehensive, evidence-based, and contextually relevant. This refinement process will involve careful consideration of the care context in the Saguenay-Lac-St-Jean (SLSJ) region, ensuring that the interventions are feasible and effective in real-world settings.

Ultimately, this methodical refinement will produce a detailed and actionable intervention program that is both theoretically sound and practically applicable (Bartholomew et al., 2016; Bartholomew and Mullen, 2011), tailored to the unique needs and vulnerabilities of families accompanying children with cancer.

#### 2.2.5 Step 5: development of the program’s implementation plan

The fifth step will involve developing a comprehensive implementation plan for The Program. This phase will be conducted to ensure that the intervention is effectively integrated into the existing healthcare framework and is sustainable over time. The goal is to identify key stakeholders, select appropriate strategies for adoption and integration, and consider factors that could facilitate or hinder the implementation process (Bartholomew et al., 2016).

The WGs will select strategies for the adoption and integration of The Program. These strategies will be informed by insights gathered from the previous steps and will be tailored to fit the specific context of the Saguenay-Lac-Saint-Jean region. We will use the (CFIR) (Carrandi et al., 2024; Damschroder et al., 2009) to guide this process. The CFIR provides a comprehensive approach to assess multiple dimensions that affect implementation, including intervention characteristics, outer and inner setting, characteristics of the individuals involved, and the implementation process itself.

We will also consider potential facilitators and barriers to implementation (Bartholomew et al., 2016; Carrandi et al., 2024). Facilitators might include existing support systems, community engagement, and available resources, while barriers could involve resistance to change, limited resources, or logistical challenges (Bartholomew et al., 2016). By identifying these factors early, we can develop strategies to address them, such as training programs to build capacity, modifying workflows to accommodate new practices, or securing additional funding to support the intervention.

A detailed timeline and action plan will be created, outlining each step of the implementation process. This plan will include specific tasks, responsible parties, deadlines, and necessary resources. Regular progress reviews and adjustments will be made to ensure the implementation stays on track and can adapt to any unforeseen challenges.

To ensure the intervention is comprehensive and effective, we will evaluate key implementation outcomes, including:

- **Intervention Characteristics**: Assessing the quality, adaptability, and complexity of the intervention to ensure it meets the needs of the target population and can be effectively integrated into existing systems.
- **Outer Setting**: Evaluating external influences such as patient needs and resources, community support, and external policies or incentives that may impact the implementation process.
- **Inner Setting**: Examining internal organizational factors, including the culture, readiness for implementation, communication networks, and resources available within the healthcare settings where the intervention will be implemented.
- **Characteristics of Individuals**: Understanding the knowledge, beliefs, and attitudes of the individuals involved in the implementation process, including health professionals and families.
- **Implementation Process**: Monitoring the stages of implementation, including planning, engaging stakeholders, executing the intervention, and evaluating its progress and outcomes.

By developing a comprehensive implementation plan using the CFIR framework and evaluating these key implementation outcomes, we will ensure that The Program is effectively integrated into the healthcare system and is sustainable and scalable. This structured approach will enable us to systematically address any challenges and leverage facilitators to enhance the overall impact of the intervention, ultimately improving the well-being and resilience of families accompanying children with cancer.

#### 2.2.6 Step 6: development of the program’s evaluation plan

The sixth step in the Intervention Mapping (IM) approach involves developing a comprehensive evaluation plan to assess the effectiveness and impact of The Program. This phase will ensure that the intervention achieves its intended outcomes and provides valuable insights for future improvements. The evaluation will focus on both process and outcome measures, employing a mixed-methods approach to capture quantitative and qualitative data.

Data collection in this specific step will include a variety of methods to ensure a comprehensive evaluation of The Program. These methods will encompass registries, clinical assessments, self-report questionnaires, and semi-structured interviews with participants and health professionals.

- **Registry**: An online registry will be used to record feasibility data, such as referral rates, eligibility rates, recruitment rates, time windows, and baseline assessment periods. Acceptability will be assessed by the Credibility Scale and the Treatment Perceptions Questionnaire (TPQ) (Sancassiani et al., 2021). TPQ will provide satisfaction information by collecting the level of agreement (5-point Likert scale, fully disagree to fully agree) regarding items measuring acceptability, suitability, tolerability, expectation of positive benefit, credibility, efficacy, appropriateness, reasonableness, justification, and discomfort.
- **Clinical Assessments and Self-Report Questionnaires**: Baseline (T1) and post-intervention (T2) clinical assessments and self-report questionnaires (Varni et al., 2007) will document family outcomes (based on step 4), including health-related quality of life, family functioning, anxiety, depression, and distress. These assessments will also evaluate the perceived security, confidence, and satisfaction with program components. Additionally, the number of objectives achieved relative to those set in the previous steps will be assessed. Medical records concerning administration of cancer treatment (baseline dose, dose reduction, delays or cancelation) data will be collected at T3 by using information tallied on the patient electronic medical record.
- **Semi-Structured Interviews**: At the end of the program (T3), semi-structured interviews will be conducted with participants and health professionals to gather in-depth qualitative data on the feasibility, fidelity, barriers, and facilitators of the intervention. Interviews will be transcribed and analyzed to identify common themes and insights.

By developing a thorough evaluation plan, we will ensure that The Program is systematically assessed for its effectiveness, feasibility, and sustainability (Bartholomew et al., 2016). This approach will provide robust evidence to support the intervention’s impact on the well-being and resilience of families accompanying children with cancer and inform future scaling and adaptation efforts.

Quantitative data will be analyzed using statistical software such as SPSS (IBM Corporation, 2017). Descriptive statistics (Tabachnick and Fidell, 2019) will summarize sociodemographic and clinical characteristics, feasibility indicators, and outcome measures. Comparative analyses (Field, 2017), such as paired t-tests or Wilcoxon signed-rank tests, will evaluate pre- and post-intervention changes. Effect sizes will be calculated to determine the magnitude of changes (Cohen, 1988). Qualitative data from interviews will be analyzed using inductive content analysis procedures (Miles et al., 2014). Themes and patterns will be identified independently by two researchers, and discrepancies will be resolved through discussion to create a common codebook. Data will be coded using software like NVivo to ensure systematic and rigorous analysis.

We will evaluate key implementation outcomes based on the CFIR, including intervention characteristics, outer setting, inner setting, characteristics of individuals, and the implementation process. These outcomes will help us understand the factors influencing the successful integration and sustainability of The Program.

The integration of quantitative and qualitative data will provide a comprehensive understanding of The Program’s effectiveness and impact. This mixed-methods approach will allow us to triangulate findings, identify discrepancies, and gain deeper insights into the intervention’s outcomes and implementation processes. Findings from the evaluation will be used to refine and improve The Program. Feedback loops will be established to ensure that insights from participants and stakeholders are incorporated into ongoing program development and enhancement.

### 2.3 Research participants

This research necessitates three distinct samples: the first comprising family members of children currently battling cancer or those who have previously faced the disease; the second consisting of adults who were diagnosed with cancer during their childhood; and the third incorporating professionals within the healthcare system who actively contribute to the care of these families. Participants from all categories will be selected using a purposive sampling strategy (Grove et al., 2013), striving for maximum variation (Creswell, 2013). This approach is underpinned by several foundational criteria: participants must (i) be at least 18 years of age, (ii) be French-speaking, (iii) reside in the Saguenay−Lac-Saint-Jean region, and (iv) possess the mental and physical capacity to engage fully in the research process. To promote equity, diversity, and inclusion, as desired in Canadian research, the selection criteria were chosen to avoid marginalizing or discriminating against individuals due to physical or mental health issues. Therefore, the decision to participate is left to the individual’s judgment based on their reality.

The constitution of each sample will be also based on specific inclusion criteria. For the family members sample—the criteria are: (i) being part of a family where a child is undergoing cancer treatment or has done so in the past, and (ii) being actively involved in supporting a child who is either currently receiving treatment or has concluded their treatment within the past 5 years.

For the adult survivors of pediatric cancer sample—the individuals must: (i) have received a cancer diagnosis during childhood, and (ii) be currently undergoing treatment or have completed it within the last 5 years.

Lastly, the healthcare professionals sample requires participants to: (i) be an integral part of a pediatric oncology care team, (ii) work directly with children affected by cancer and their families, and (iii) hold a professional role pertinent to the program under study.

This meticulous selection process ensures a comprehensive and empathetic understanding of the varied experiences and challenges faced by each distinct group, ultimately enriching the research outcomes and their potential applications.

### 2.4 Ethical considerations

Ethics approval for this study was obtained from the Centre intégré universitaire de santé et de services sociaux du Saguenay-Lac-Saint-Jean. This clinical study will be conducted in accordance with applicable Health Canada regulations, International Conference on Harmonization (ICH) guidelines on current Good Clinical Practice (GCP), and the Declaration of Helsinki. All participants will be given detailed oral and written information about the study. Consent forms describing the study procedures and risks in detail will be given to each participant and written documentation of informed consent will be required prior to starting study protocol.

In adherence to the best ethical practices for the preservation and archiving of research data, all research materials, including signed consent forms, completed questionnaires, and any other essential documents, will be meticulously stored in a secure, fire-resistant filing cabinet, located within a restricted-access office at UQAC. This office will be assigned to one of the lead researchers, thereby ensuring that only authorized personnel can access it, in order to maintain the confidentiality and integrity of the data.

Moreover, to prevent any form of data loss, and then compromise confidentiality, a digital copy of the documents will be created. These files will be encrypted and saved on a secure server at UQAC, with scheduled regular backups to ensure data preservation. These digital security measures are crucial in preventing data losses due to hardware failures, human errors, or natural disasters. The data retention period will be in compliance with ethical and regulatory standards, with a planned retention period of 5 years following the conclusion of the study. Beyond this period, all data will be securely destroyed to ensure that no sensitive or personal information can be retrieved subsequently. This destruction will be conducted under the supervision of an official, and a report confirming the secure destruction of data will be compiled and kept in the project’s archives. It’s also essential to specify that all procedures for data preservation, security, and destruction will be performed in strict compliance with privacy protection regulations and industry ethical guidelines, thereby ensuring the rights and confidentiality of research participants are respected at every stage of the process.

## 3 Discussion

This protocol is grounded in extensive scientific literature highlighting the multifaceted and challenging nature of the family experience in pediatric oncology. Families dealing with pediatric cancer face significant vulnerabilities and transformations, with these challenges exacerbated by geographic distance from tertiary care centers (Walling et al., 2019; Van Shoors et al., 2015; Wilson et al., 2016). Despite these unique challenges, the experience of families in remote areas remains underexplored, and the care and services provided to them are often inconsistent (Walling et al., 2019).

Parents of children with cancer are particularly vulnerable psychosocially, and those with fewer resources to support their resilience are even more at risk (Rosenberg et al., 2014). Many authors have emphasized the importance of supporting the resilience of these families (McCubbin et al., 2002; Van Shoors et al., 2015; Masera et al., 2013), yet there is a notable gap in the literature regarding interventions specifically designed for families living far from tertiary care centers. This protocol aims to address this gap by supporting the resilience process of these children and their families to prevent and mitigate the potential biopsychosocial and spiritual problems they may face.

As Koumarianou et al. highlight, “Psychosocial interventions in families of children with cancer are considered an effective way of empowering family members to tackle the complex hurdles they face. The ability of parents to develop adaptive coping strategies during the child’s treatment is not only important to their own mental and physical health, but also to their child’s well-being and long-term adjustment with the disease” (Koumarianou et al., 2021) (p. 103).

The systematic development of this program, using a collaborative, interdisciplinary, and ecosystemic approach that integrates theoretical frameworks, empirical evidence, and practical insights from stakeholders, is innovative. It aims to meet the multifaceted needs of families accompanying children with cancer in the Saguenay-Lac-Saint-Jean region. As highlighted by Toruner and Altay (2018) and Snaman et al. (2018), family-centered care is imperative in contemporary pediatric oncology care. However, it is not always clearly understood and integrated into different care settings. The collaborative approach with children, families, and healthcare team members in this protocol is designed to facilitate this integration.

Methodologically, the logical models and matrices associated with the program in this study will offer rigorous syntheses of the vulnerability issues faced by these children and their families, the strengthening of their resilience process, and the necessary multidisciplinary strategies to ultimately improve their well-being and quality of life. These models will serve as valuable references for researchers, healthcare professionals, and policymakers who seek to better understand the realities of these families and apply relevant clinical interventions. The structured and modeled program development approach should also enhance the feasibility, adoption, and ultimately the implementation and evaluation of the program, as noted by Kok et al. (2014, 2017): “The Intervention Mapping protocol helps program planners to optimize chances for effectiveness” (Kok et al., 2014) (p. 105).

In conclusion, this protocol represents a significant step toward developing an evidence-based, contextually relevant program that addresses the unique needs of families accompanying children with cancer in remote areas. The integration of family resilience frameworks, stakeholder input, and rigorous evaluation methods ensures that the program is well-positioned to improve the well-being and resilience of these families, ultimately enhancing their ability to cope with the challenges of pediatric cancer.
